# Cosmetic Potential of Pigments Extracts from the Marine Cyanobacterium *Cyanobium* sp.

**DOI:** 10.3390/md20080481

**Published:** 2022-07-27

**Authors:** Fernando Pagels, Cíntia Almeida, Vitor Vasconcelos, A. Catarina Guedes

**Affiliations:** 1 CIIMAR/CIMAR-LA—Interdisciplinary Centre of Marine and Environmental Research, University of Porto, Novo Edifício do Terminal de Cruzeiros de Leixões, Avenida General Norton de Matos, s/n, 4450-208 Matosinhos, Portugal; fernandopagels@gmail.com (F.P.); acatarinaguedes@gmail.com (A.C.G.); 2 FCUP—Faculty of Science, University of Porto, Rua do Campo Alegre, s/n, 4169-007 Porto, Portugal; 3 ISS—Ínclita Seaweed Solutions, Novo Edifício do Terminal de Cruzeiros de Leixões, Avenida General Norton de Matos, s/n, 4450-208 Matosinhos, Portugal; cin_almeida@hotmail.com

**Keywords:** carotenoids, phycobiliproteins, antioxidant, hyaluronidase, accelerated stability, serum formulation

## Abstract

The current mindset in the cosmetics market about sustainable ingredients had increased the search for new sources of natural active ingredients. Cyanobacteria are a great source of functional ingredients for cosmetics, as a producer of pigments with described bioactive potential (carotenoids and phycobiliproteins). This work aimed to evaluate the cosmetic potential of marine cyanobacterium *Cyanobium* sp. pigment-targeted extracts (carotenoids and phycobiliproteins), evaluating their in vitro safety through cytotoxicity assays, cosmetic-related enzyme inhibition, ingredient stability, and putative product (serum formulation). Results showed no cytotoxicity from the extracts in skin-related cell lines. Carotenoid extract showed anti-hyaluronidase capacity (IC_50_ = 108.74 ± 5.74 mg mL^−1^) and phycobiliprotein extract showed anti-hyaluronidase and anti-collagenase capacity (IC_50_ = 67.25 ± 1.18 and 582.82 ± 56.99 mg mL^−1^, respectively). Regarding ingredient and serum stability, both ingredients showed higher stability at low-temperature conditions, and it was possible to maintain the pigment content and bioactive capacity stable during the tested period, although in higher temperatures the product was degraded in a week. As a major conclusion, both extracts can be potential natural and sustainable ingredients for cosmetic uses, with relatively simple formulation and storage, and can be promising natural anti-aging ingredients due to their bioactive capacity.

## 1. Introduction

The use of natural ingredients for aesthetic and health-enhancing applications in popular and homemade recipes and without scientific bases existed long before today’s concept of cosmetics. Thus, for a long time, cosmetics were made from mineral materials and herbal pastes and oils [1]. Nowadays, the movement toward green packaging, recycling, and environmental literacy has contributed to a revolution in the demands of the cosmetics industry and the rescue of already established suppliers of bioactive products, plants, and algae [2]. From the formulation to the packaging material, the cosmetic industry has been moving toward adaptation to greener manufacturing [3].

This dynamic industry is now looking for new opportunities and new goods with features other than beauty; a new segment of the industry, called cosmeceuticals, started with the use of cosmetics for health purposes [2]. Although natural origin does not necessarily mean “healthy”, it is nevertheless true that the compounds and extracts used in natural cosmetics are also potentially beneficial to health [2].

The use of cyanobacteria as a source of ingredients achieves even greater potential with the idea of cosmeceuticals. These organisms can be a source of compounds with narrowly defined potential for health applications, such as fatty acids, polyphenols, peptides, polysaccharides, and pigments [4]. When it comes to cyanobacterial pigments in special carotenoids and phycobiliproteins, it is possible to combine their bioactive capacity, varied colors, and cosmetic enhancement characteristics (e.g., moisturizer, stabilizing agents) and draw interest from the natural cosmetic industry [5,6]. The use of extracts overcomes one of the biggest concerns of the industry, which is product stability. As these complex matrices contain other compounds besides pigments, they often increase not only the stability but also the useful life of the pigments referred to above (e.g., polyphenols) [6].

Carotenoids can be included in sunscreen, anti-ageing, and antioxidant formulations due to their high-efficiency antioxidant capacity. These compounds, inside cyanobacteria cells, are responsible for capturing exciding energy from the photosynthetic metabolism, mitigating harmful effects and cell damage [7]. Once extracted, these compounds can be used for a similar purpose in human skin, where prolonged exposure to sunlight (UV radiation and high light intensity) contributes to potential damage to cells [8,9].

A similar thought can be applied to phycobiliproteins, as they can act as functional additives, since they have been associated with a variety of bioactivities, such as antioxidants, antivirals, and antimicrobials and antitumors, among others [10]. Moreover, these compounds can be used as natural dyes to mitigate toxicity and damage to the skin, as well as allergy to synthetic dyes [11]. These blue hydrophilic pigments can be used in skincare products, particularly creams and lotions, due to their water solubility.

While there is a great diversity of cyanobacteria species and their biochemical profile, *Arthrospira platensis* (spirulina) is still the main choice for cosmetic usage. Yet, other cyanobacteria species have been suggested in fundamental research. In addition, most studies concerning the potential of cyanobacteria in cosmetics are focused on chemical characterization and bioactivity prospection, whereas the real applicability as an ingredient in a final product has been kept in the industry and rarely described in academic research.

This research focused on marine cyanobacterium *Cyanobium* sp. LEGE 06113, which appears to be an effective strain for industrial use due to its morphological characteristics and chemical composition. This strain is a fast-growing unicellular organism with a highly adaptable metabolism and response to light and with great production of pigments, both carotenoids and phycobiliproteins [12]. Moreover, the extracts from *Cyanobium* sp. have been proposed as a great potential source of antioxidant and anti-inflammatory capacity and a sustainable option as a cyanobacteria-based bioprocess, as from the same biomass both a carotenoid- and a phycobiliprotein-rich extract can be obtained in a successive extraction method [13] following the principle of environmental and economic sustainability.

Therefore, this work aims to evaluate the cosmetic potential of *Cyanobium* sp. pigment-targeted extracts, evaluating their in vitro safety through cytotoxicity assays and cosmetic-related enzymes inhibition. Furthermore, to assess the real applicability as a cosmetic ingredient, the formulation of an ingredient and its application in a product (serum) was performed, considering stability as the main parameter for the assessment.

## 2. Results

The cosmetic potential of *Cyanobium* sp. pigments was evaluated following a structured line of thought that starts from the assessment of the potential of the extract as a cosmetic ingredient. Then the extracts were proposed as an active part of a cosmetic ingredient, being evaluated in terms of bioactive capacity and stability. Finally, as proof of concept, the ingredient was included in an end product (skin serum) to assess the compatibility, bioactive capacity, and stability of the ingredient with a more complex formulation.

### 2.1. Extract Cytotoxicity

**Figure 1:**
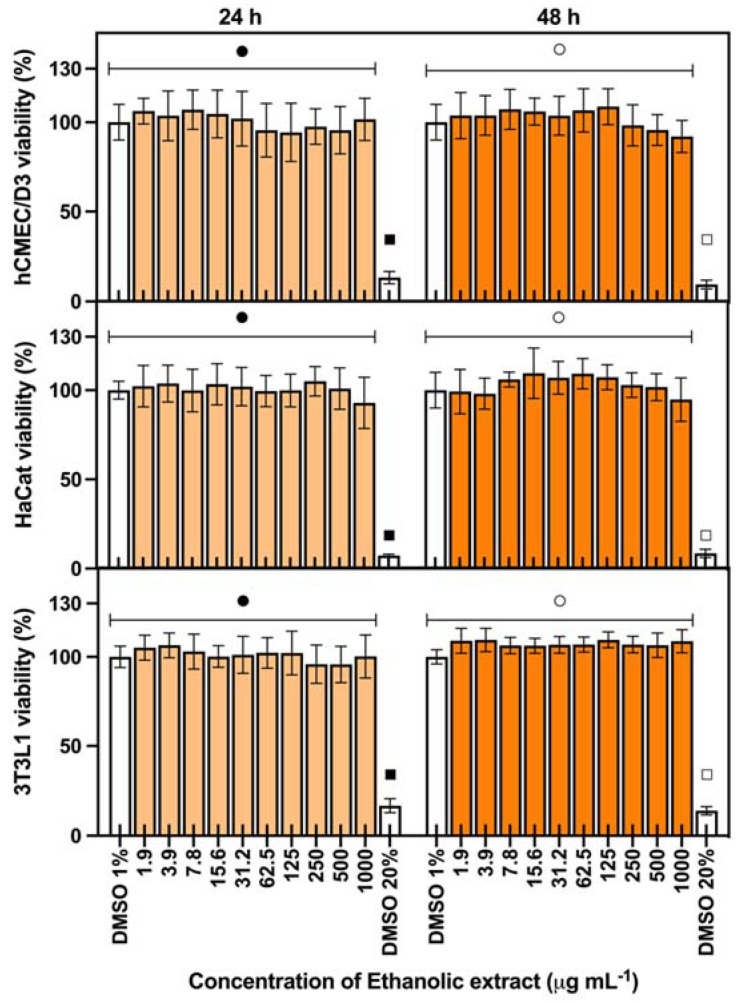
Cytotoxicity of *Cyanobium* sp. ethanolic extract in skin-related cell lines: endothelial (hCMEC/D3), epithelial (HaCat), and fibroblast (3T3L1) exposed during 24 and 48 h (average ± standard deviation, *n* = 3). Different symbols in the same graphic for each exposure time mean significant differences between the extract concentration and the negative control with DMSO 1% (*p* < 0.05). DMSO 20% is the positive control for the assay.

**Figure 2:**
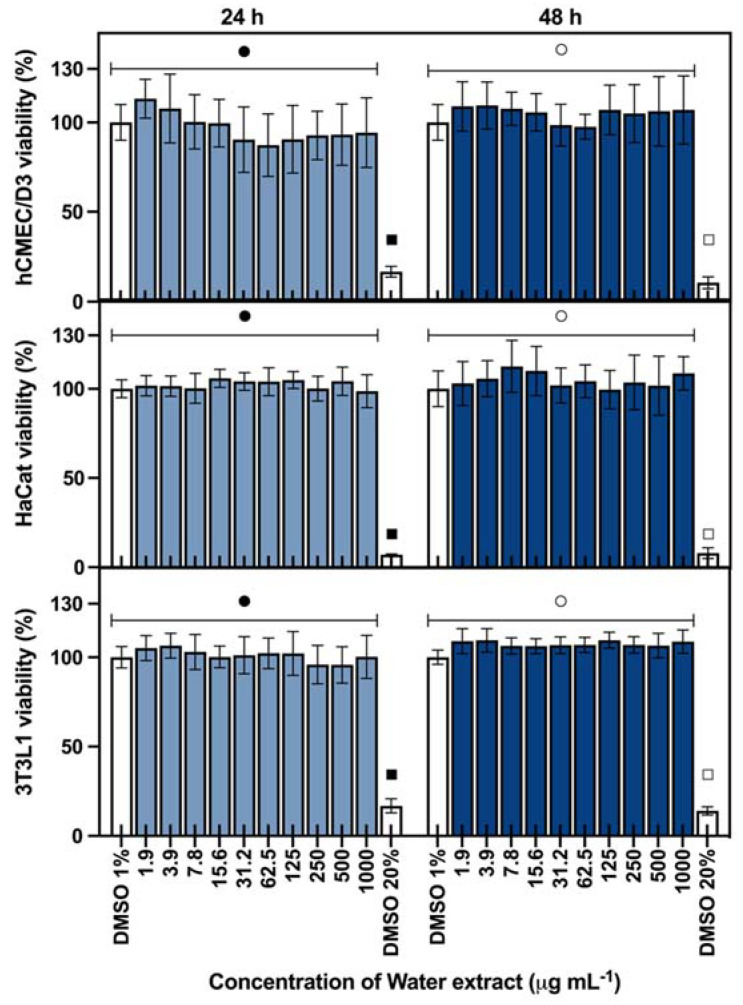
Cytotoxicity of *Cyanobium* sp. water extract in skin-related cell lines: endothelial (hCMEC/D3), epithelial (HaCat), and fibroblast (3T3L1) exposed during 24 and 48 h (average ± standard deviation, *n* = 3). Different symbols in the same graphic for each exposure time mean significant differences between the extract concentration and the negative control with DMSO 1% (*p* < 0.05). DMSO 20% is the positive control for the assay.

Considering a cosmeceutical approach for the extracts from *Cyanobium* sp., their cytotoxicity was evaluated in three skin-related cell lines, keratinocytes (HaCat), fibroblasts (3T3L1), and endothelial (hCMEC/D3) cells. The results are shown in Figure 1 for ethanolic extract and Figure 2 for water extract. Both ethanolic and water extracts showed no cytotoxic effects on the evaluated cell lines, with no significant differences from the control (*p* > 0.05), for both 24 and 48 h of exposition in concentrations up to 1000 μg mL^−1^, meaning that the extracts can be safe for cosmetic use, with no toxicity to skin-related cell lines.

### 2.2. Enzymatic Activity

Skin aging is one of the most meaningful processes that cosmetics attempt to address. The most noticeable changes are skin dryness, decreased elasticity, fine wrinkles, and skin changes that result in expression lines [14]. In cosmetics, evaluation of enzymatic activity can add value and increase the potential of the ingredient. The inhibition of enzymes such as hyaluronidase, collagenase, tyrosinase, and elastase is a goal for new products regarding the anti-aging application. The inhibition of hyaluronidase, elastase, and collagenase is mainly related to a decrease in wrinkles and enhancement of skin elasticity, whereas the inhibition of tyrosinase is related to skin whitening and anti-melanogenesis treatments.

Here both extracts were evaluated to be used as inhibitors of such enzymes. Ethanolic extract only was able to inhibit hyaluronidase, with an inhibitory concentration of 108.74 ± 5.74 μg mL^−1^ (IC_50_), whereas the water extract was able to inhibit both hyaluronidase and collagenase, with an IC_50_ of 67.25 ± 1.18 and 582.82 ± 56.9, respectively. Noteworthy is that the water extract was 1.6-fold better than the ethanolic extract in the hyaluronidase inhibition. Finally, no inhibition of tyrosinase and elastase was found in concentrations up to 1000 μg mL^−1^ in any of the two extracts. Due to the higher potential as anti-hyaluronidase components, in comparison to the potential as anti-collagenase, only this enzymatic inhibition activity was considered for further evaluation. As a proof of concept, a water extract from *Camellia sinensis* (green tea) was obtained using the same setup from *Cyanobium* sp. first extraction, as it is a known ingredient for anti-aging formulations [15]. The IC_50_ of green tea for hyaluronidase was 122.19 ± 7.64 μg mL^−1^, with no statistical differences to the ethanolic extract of *Cyanobium* sp. and 1.8-fold higher than the water extract.

### 2.3. Cyanobium sp. Cosmetic Ingredients

For better incorporation in cosmetic formulations, two ingredients composed of the active extract and a liquid vehicle (glycerol for the water extract and linseed oil for the ethanolic extract) were proposed. The ingredients were then evaluated in terms of their physical–chemical characterization, bioactive capacity, and stability. Moreover, many commercial ingredients include a preservative (antioxidant) to prevent degradation of the active ingredient (extract). Taking that into account, this study also evaluated the compatibility of the ingredient with several antioxidants to increase its stability. The results of these assessments are described in the following sections.

#### 2.3.1. Ingredient Characterization

The general composition of a cosmetic includes an active ingredient and excipients (vehicle, thickening agents, and additives) [11]. The active ingredients are the main components of a cosmetic product that are responsible for its function, in this case, pigment extracts. Furthermore, excipients are all ingredients that do not serve a specific function as an active ingredient, such as the active ingredient’s vehicle, thickening agents, and additives (preservatives, colorants, and perfumes). To increase the stability of the extracts and make their incorporation in different formulations possible, the ethanolic extract was added to linseed oil (ethanolic ingredient) and the water extract was added to 80% glycerol (water ingredient). The physical and chemical characteristics of the ingredients are presented in Table 1. The bioactive capacity (antioxidant and anti-hyaluronidase) and the content of the total pigment were also measured to control the potential of the ingredients before and after the stability assays.

#### 2.3.2. Hot–Cold Ingredient Stability

**Figure 3:**
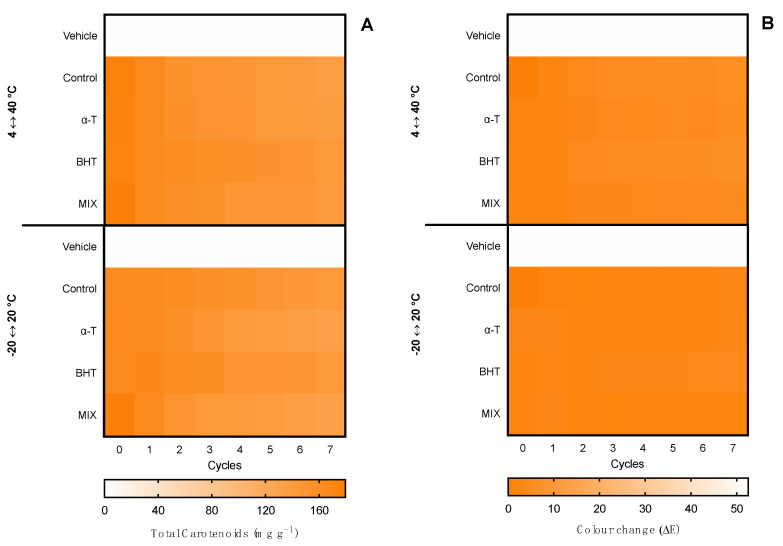
Effect of hot–cold cycles (4 °C ↔ 40 °C and −20 °C ↔ 20 °C) in (**A**) total carotenoids and (**B**) color change (ΔE) of ethanolic ingredients supplemented with commercial antioxidants: α-tocopherol (α-T), BHT, or a mixture of both (1:1; MIX). The vehicle represents linseed oil and control is the ingredient without supplement.

**Figure 4:**
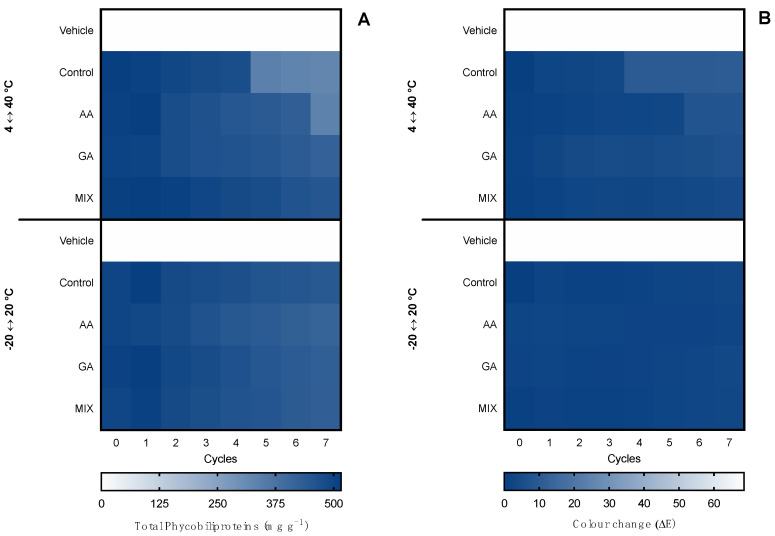
Effect of hot–cold cycles (4 °C ↔ 40 °C and −20 °C ↔ 20 °C) in (**A**) total phycobiliproteins and (**B**) color change (ΔE) of water ingredients supplemented with commercial antioxidants: ascorbic acid (AA), gallic acid (GA), or a mixture of both (1:1; MIX). The vehicle represents glycerol (80%) and control is the ingredient without supplement.

**Figure 5:**
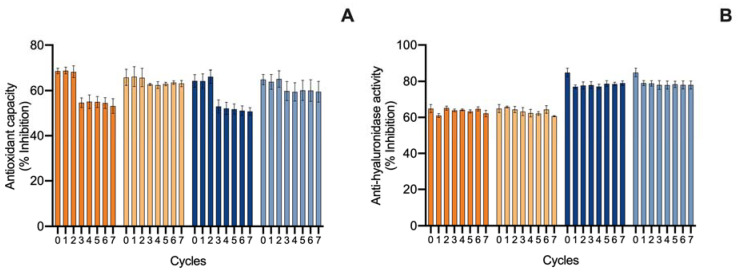
Effect of hot–cold cycles (4 °C ↔ 40 °C and −20 °C ↔ 20 °C) on the (**A**) ABTS^•+^ scavenging ability and (**B**) hyaluronidase inhibition capacity (% inhibition; average ± standard deviation, *n* = 3) of the ethanolic (E) and water (W) ingredient. Colors of lines indicate different conditions: E 4 °C ↔ 40 °C (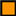); E −20 °C ↔ 20 °C (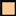); W 4 °C ↔ 40 °C (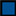); W −20 °C ↔ 20 °C (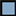).

The first evaluation of ingredient stability was performed in hot–cold cycles for 14 days in −20 ↔ 20 °C and 4 ↔ 40 °C cycles. The extracts were evaluated in terms of total pigments, color change (ΔE), and bioactive capacity. Moreover, the supplementation with commercial antioxidants (α-tocopherol, BHT, and a mixture of both (1:1) for the ethanolic ingredient and ascorbic acid, gallic acid, and a mixture of both (1:1) for the water ingredient) was tested for compatibility and antioxidant effects in the thermic treatments. Results for the ethanolic ingredient are shown in Figure 3 and for the water ingredient in Figure 4. Moreover, due to the influence of antioxidant supplements in the bioactive capacity assays, only the control condition was evaluated for both extracts (Figure 5).

Regarding the ethanolic ingredient, both the content of carotenoids and color were kept stable in both −20 °C ↔ 20 °C and 4 °C ↔ 40 °C treatments, with no statistical differences regardless of the antioxidant supplementation or the number of cycles, as observed by the homogeneous color gradient present in Figure 3.

When it came to the water ingredient, the content of phycobiliproteins was kept stable in the −20 °C ↔ 20 °C treatment, with no statistical differences regardless of the antioxidant supplementation or the number of cycles. With the 4 °C ↔ 40 °C treatment, a reduction was observed from cycle 5 in the control condition without antioxidant supplementation and in cycle 7 in the ingredient supplemented with ascorbic acid.

Regarding the color, in the −20 °C ↔ 20 °C treatment no perceptible color change was observed in the seven cycles (ΔE < 2.0), whereas in the 4 °C ↔ 40 °C treatment, a linear change was observed. From cycle 2, the color had a perceptible change (ΔE > 2.0), with less accentuated change in the ingredients supplemented with gallic acid and the mixture of antioxidants. The maximum color change was found in cycles 6 and 7, with an ΔE ≈ 7.0, which is still an acceptable color change in stability assays.

Finally, regarding the bioactive capacity (antioxidant and anti-hyaluronidase) of the ingredients (Figure 5), both of them were stable in the −20 °C ↔ 20 °C treatment and the anti-hyaluronidase activity was stable even under the 4 °C ↔ 40 °C treatment. However, in the 4 °C ↔ 40 °C treatment, both ingredients had a loss of antioxidant capacity, translated by a decrease in the inhibition of ABTS^•+^ from the third cycle by 0.2-fold in the ethanolic ingredient and by 0.3-fold in the water ingredient. On the other hand, the antioxidant capacity remained present, with an inhibition higher than 50%.

#### 2.3.3. Accelerated Ingredient Stability

**Figure 6:**
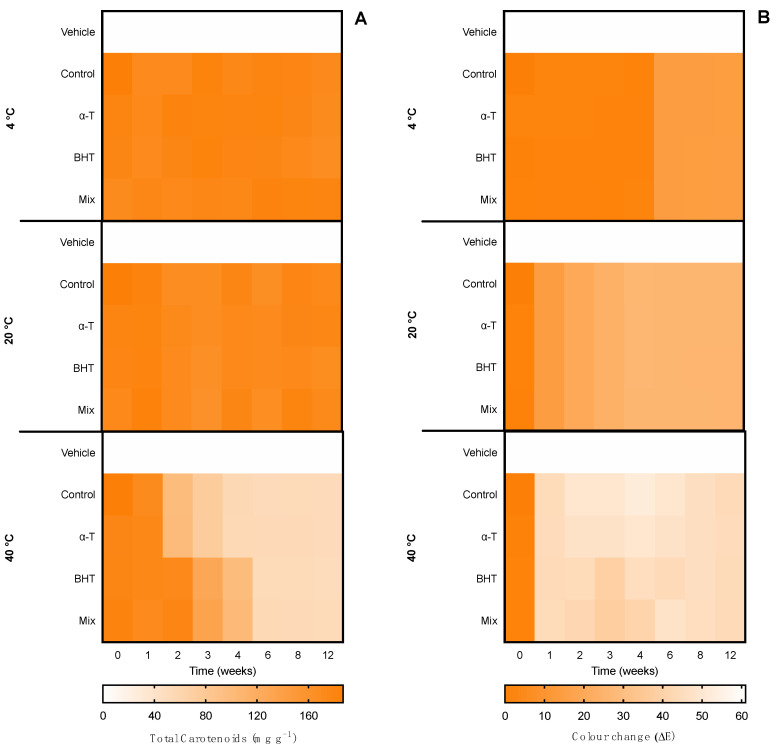
Effect of accelerated conditions (4 °C, 20 °C, and 40 °C) in (**A**) total carotenoids and (**B**) color change (ΔE) of ethanolic ingredients supplemented with commercial antioxidants: α-tocopherol (α-T), BHT, or a mixture of both (1:1; MIX). The vehicle represents linseed oil and control is the ingredient without supplement.

**Figure 7:**
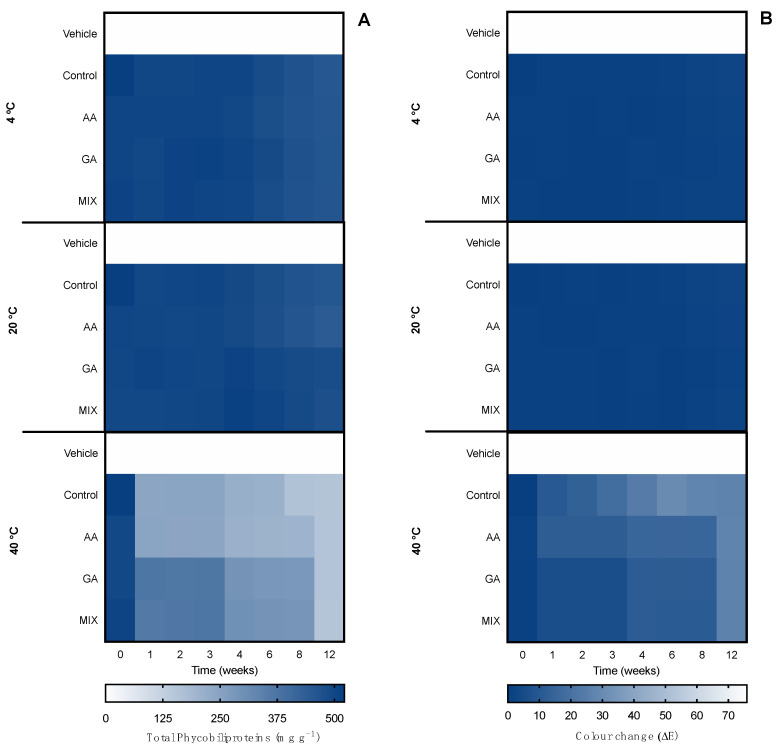
Effect of accelerated conditions (4 °C, 20 °C, and 40 °C) in (**A**) total phycobiliproteins and (**B**) color change (ΔE) of water ingredients supplemented with commercial antioxidants: ascorbic acid (AA), gallic acid (GA), or a mixture of both (1:1; MIX). The vehicle represents glycerol (80%) and control is the ingredient without supplement.

**Figure 8:**
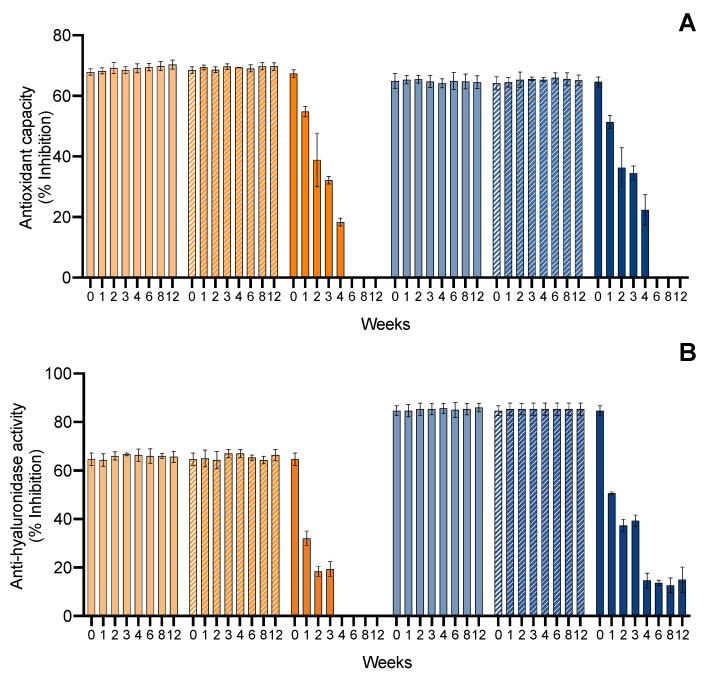
Effect of accelerated conditions (4 °C, 20 °C, and 40 °C) on the (**A**) ABTS^•+^ scavenging ability and (**B**) hyaluronidase inhibition activity (% inhibition; average ± standard deviation, *n* = 3) of ethanolic (E) and water (W) ingredients. Colors of lines indicates different conditions: E 4 °C (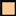); E 20 °C (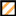); E 40 °C (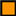); W 4 °C (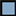); W 20 °C (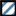); W 40 °C (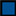).

The second evaluation of the ingredient stability involved an accelerated stability assay for 12 weeks, which represented long-exposure effects in the ingredient. Such tests are demanded by quality-control agencies, such as ISO/TR 18811/2018 and European Regulation EC 1223/2009. The ingredients were monitored over time for pigment content, color change, and bioactive capacity. Results are shown in Figure 6 for the ethanolic ingredient and Figure 7 for the water ingredient. Due to the influence of antioxidant supplements in the bioactive capacity assays, only the control condition was evaluated for both ingredients (Figure 8).

Regarding the ethanolic ingredient, the content of carotenoids was kept stable for 12 weeks under 4 °C and 20 °C treatments, with no statistical differences regardless of the antioxidant supplementation or the number of cycles. On the other hand, in the 40 °C treatment, both control and α-tocopherol supplemented ingredients were degraded from week 2, whereas the ingredients supplemented with BHT and a mix of antioxidants resisted two weeks longer. From week 4, all conditions were degraded, reaching the minimum of ca. 50 mg g^−1^ of carotenoid, 3.6-fold less than the initial content.

When it comes to the color, all temperatures led to changes in the ingredient color. The 4 °C treatment led to a color change regardless of antioxidant supplementation. For the first month, no perceptible change was found (ΔE < 2.0); from week 6 to 12, the color went from bright green to dark yellow with a ΔE ≈ 14.0. At 20 °C a greater color change was observed from week 1 (ΔE ≈ 14.0), stabilizing with ΔE ≈ 26.0 in 6 weeks, regardless of the antioxidant supplementation. The optical equivalence changed from bright green to yellow, probably meaning a chlorophyll bleaching, with no change in carotenoid content. At 40 °C, the color completely changed from green to yellow and then to brown in less than a month, resulting in a ΔE > 40.0.

When it came to the water ingredient, the content of phycobiliproteins was also stable during the 12 weeks at 4 °C and 20 °C. At 40 °C, the content decreased depending on the antioxidant supplementation. From week 1, the control ingredient and the one supplemented with ascorbic acid started to degrade. From week 4, all ingredients started to degrade, equally reaching an average of ca. 150 mg g^−1^ in week 12, representing a reduction of 70% of the pigment content.

A similar pattern was found in the color evaluation. No changes were found at 4 °C and 20 °C, with an ΔE < 1.0 until week 6 and 1.0 < ΔE < 2.0 from until week 12. At 40 °C, from week 1 to 3, the control and ascorbic acid-supplemented ingredients had perceptible color changes ΔE > 10.0, whereas ingredients supplemented with gallic acid and a mix of antioxidants had a less accentuated change (ΔE ≈ 5.0). From week 4, all ingredients had perceptible differences, as well as at week 12 (ΔE ≈ 26.0).

Finally, regarding the bioactive capacity of the ingredients (Figure 8), both had a stable antioxidant capacity in the 4 °C and 20 °C treatments. However, at 40 °C, both ingredients had a loss of antioxidant capacity, translated by a decrease in the inhibition of ABTS^•+^ from week 1, with a continuous decrease until week 4. After this, no significative inhibition was observed, meaning a loss of the antioxidant capacity of the ingredients. When it came to the anti-hyaluronidase activity, a similar pattern was found at 4 °C and 20 °C treatments, where both ingredients were stable, whereas at 40 °C, the ethanolic ingredient lost activity by week 4, and the water ingredient had a reduction from 80 to 15% of hyaluronidase inhibition by week 4.

### 2.4. Serum Formulation

#### 2.4.1. Formulation Characterization

The major goal of applying a natural extract in cosmetics is to develop a formulation using the active ingredient. Here a putative formulation was an attempt based on a skin serum described by Chowjarean et al. [16] with the addition of the concentrated ingredient: 3% of vehicle and a final extract concentration of 5 mg g^−1^. The ethanolic ingredient was introduced into the ethanolic serum and the water ingredient into the water serum. The supplementation of antioxidants was also performed as before: α-tocopherol, BHT, or a mixture of both (1:1) for the ethanolic serum and ascorbic acid, gallic acid, or a mixture of both (1:1) for the water serum. The physical and chemical characteristics of the formulated serum are presented in Table 2. The bioactive capacity and the content of the total pigment were also measured as the control of the serum before and after the stability assays. Noteworthy is that the physical–chemical characteristics were similar in both serums, although there was a phase separation in the ethanolic serum subjected to a centrifugation process. This phase separation can be easily homogenized by manual shaking, which is similar to bi-phasic products in cosmetics. In addition, the final color of the serum was very similar to the one found in the ingredient, with a similar pigment content and antioxidant capacity.

#### 2.4.2. Serum Hot–Cold Stability

**Figure 9:**
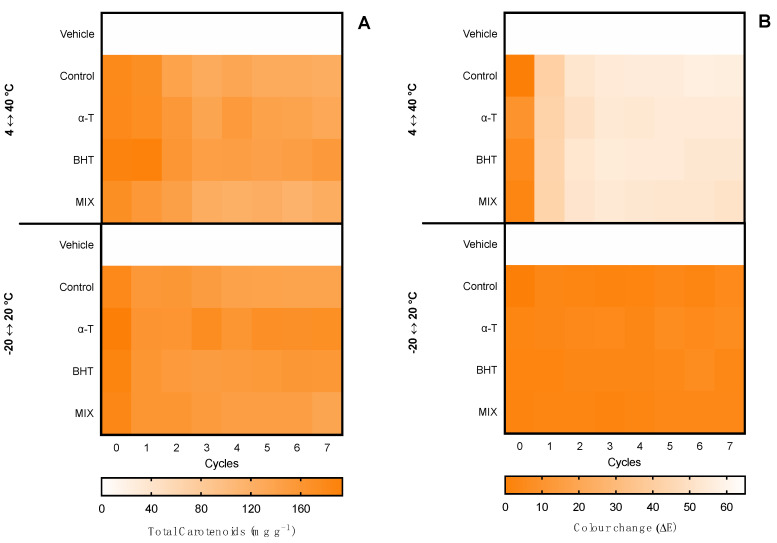
Effect of hot–cold cycles (4 °C ↔ 40 °C and −20 °C ↔ 20 °C) in (**A**) total carotenoids and (**B**) color change (ΔE) of the ethanolic serum supplemented with commercial antioxidants: α-tocopherol (α-T), BHT, or a mixture of both (1:1; MIX). The vehicle represents a serum with linseed oil and control is the serum without supplements.

**Figure 10:**
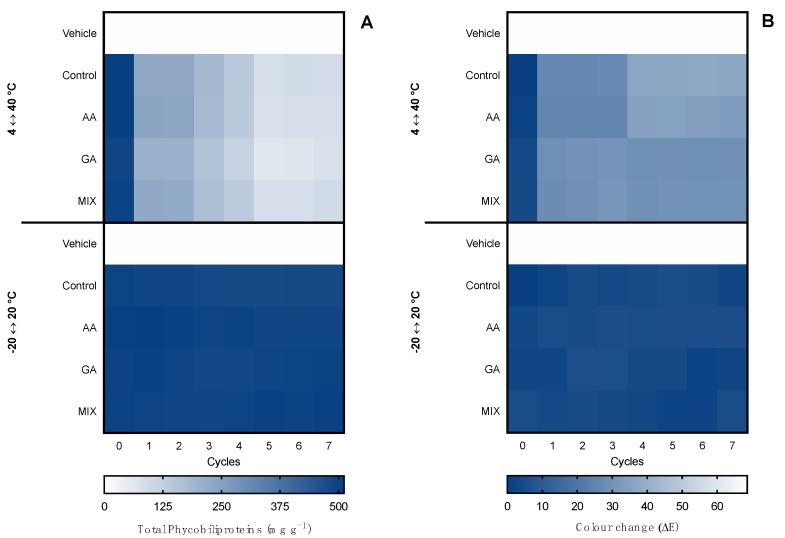
Effect of hot–cold cycles (4 °C ↔ 40 °C and −20 °C ↔ 20 °C) in (**A**) total phycobiliproteins and (**B**) color change (ΔE) of the water serum supplemented with commercial antioxidants: ascorbic acid (AA), gallic acid (GA) or a mixture of both (1:1; MIX). The vehicle represents a serum with glycerol (80%) and control is the serum without supplements.

**Figure 11:**
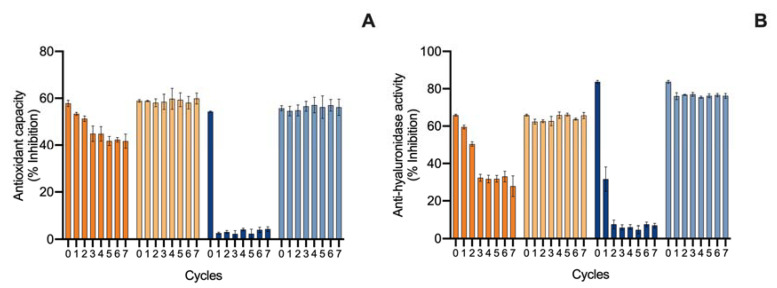
Effect of hot–cold cycles (4 °C ↔ 40 °C and −20 °C ↔ 20 °C) on the (**A**) ABTS^•+^ scavenging ability and (**B**) hyaluronidase inhibition activity (% inhibition; average ± standard deviation, *n* = 3) of the ethanolic serum (E) and water serum (W) extract ingredient. Colors of lines indicate different conditions: E 4 °C ↔ 40 °C (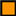); E −20 °C ↔ 20 °C (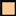); W 4 °C ↔ 40 °C (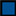); W −20 °C ↔ 20 °C (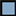).

In a similar method to that performed with the ingredient, the two formulated serums were subjected to stability tests using hot–cold cycles and temperature-accelerated stability for 12 weeks. Results for the hot–cold stability assay for the ethanolic serum are shown in Figure 9 and for the water serum are shown in Figure 10. Again, due to the influence of antioxidant supplements on the bioactive capacity assays, only the control condition was evaluated for both serums (Figure 11).

The ethanolic serum subjected to −20 °C ↔ 20 °C had a slight reduction in carotenoid content from cycle 1 of about 20%. In the one supplemented with a mixture of antioxidants, a more accentuated loss was observed (ca. 30%). When subjected to 4 °C ↔ 40 °C cycles, the content was slightly reduced in cycle 3 by 25% in the control serum and the serums supplemented with α-tocopherol and a mixture of antioxidants. The loss reached 40% by cycle 7 in the control and α-tocopherol serums, and 50% in the serums supplemented with the mixture of antioxidants. BHT supplementation protected the degradation, with a reduction of only 25% by cycle 7.

Regarding the color, no statistical differences were found in the serums subjected to −20 °C ↔ 20 °C, whereas a big color change was found in the ones subjected to 4 °C ↔ 40 °C cycles. In the 4 °C ↔ 40 °C treatment, a ΔE ≈ 35.0 was observed from cycle 1 regardless of the antioxidant supplementation, reaching ΔE > 40.0 from cycle 2. Optimal equivalence went from bright green to brown.

No statistical differences were observed in the pigment content of the water serums subjected to −20 °C ↔ 20 °C cycles for all seven evaluated cycles. On the other hand, the ones subjected to 4 °C ↔ 40 °C had a huge reduction in the content of about 50% from cycle 1, and reached a loss of 90% of pigments in cycle 5. In a similar trend, no changes were observed in the color of the water serums in the −20 °C ↔ 20 °C cycles, whereas a greater difference was found in the 4 °C ↔ 40 °C cycles. From cycle 1, the control serum and the one supplemented with ascorbic acid had a ΔE ≈ 20.0, whereas the ones with gallic acid and the mixture of antioxidants had a ΔE ≈ 30.0. From cycles 4 to 7, the color was stable but very different from the original—the control serum and the one with ascorbic acid had a ΔE ≈ 35.0, and the ones with gallic acid and the mixture of antioxidants had a ΔE ≈ 30.0. The optical equivalence went from blue to grey.

Finally, regarding the bioactive capacity of the serums (Figure 11), both were kept stable in the −20 °C ↔ 20 °C cycles. Under the 4 °C ↔ 40 °C cycles, the ethanolic serum had a reduction in the antioxidant capacity, with a decrease of inhibition power by 1.3-fold in cycle 3, whereas the water serum showed no significant antioxidant capacity (<5%) from cycle 1. Regarding the anti-hyaluronidase activity, the same accentuated reduction in cycle 3 was observed: The ethanolic serum had a reduction of 2.0-fold and the water serum lost the anti-hyaluronidase activity by cycle 2 (<7%).

#### 2.4.3. Serum Accelerated Stability

**Figure 12:**
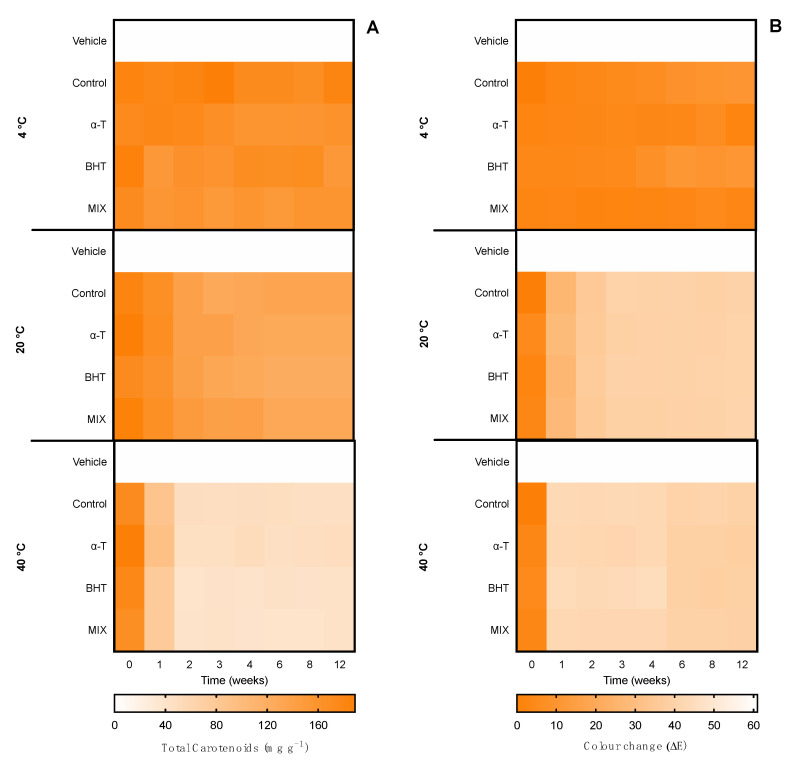
Effect of accelerated conditions (4 °C, 20 °C, and 40 °C) in (**A**) total carotenoids and (**B**) color change (ΔE) of the ethanolic serum supplemented with commercial antioxidants: α-tocopherol (α-T), BHT, or a mixture of both (1:1; MIX). The vehicle represents a serum with linseed oil and control is the serum without supplements.

**Figure 13:**
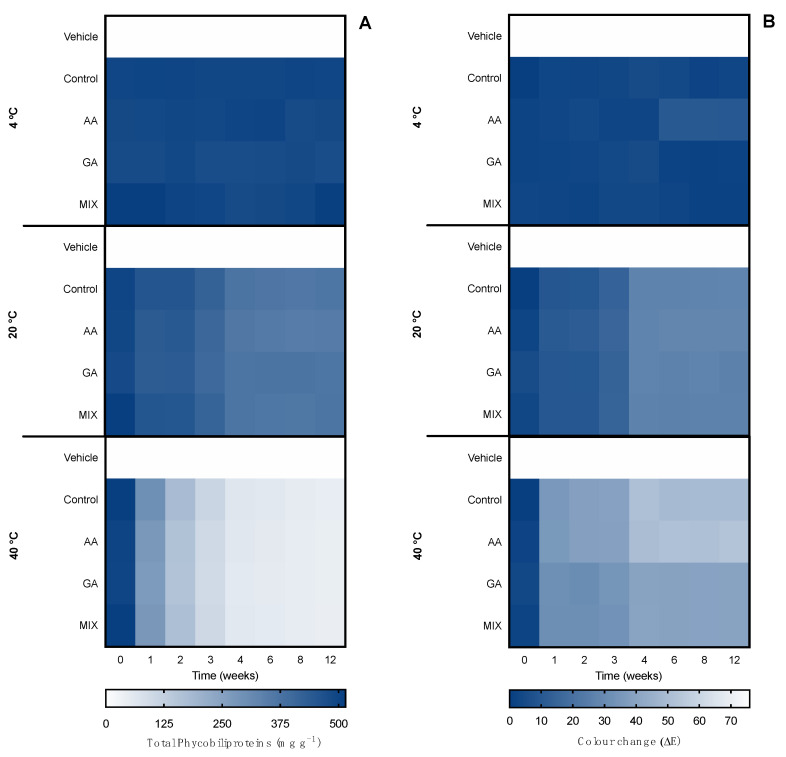
Effect of accelerated conditions (4 °C, 20 °C, and 40 °C) in (**A**) total phycobiliproteins and (**B**) color change (ΔE) of the water serum supplemented with commercial antioxidants: ascorbic acid (AA), gallic acid (GA), or a mixture of both (1:1; MIX). The vehicle represents a serum with glycerol (80%) and control is the serum without supplements.

**Figure 14:**
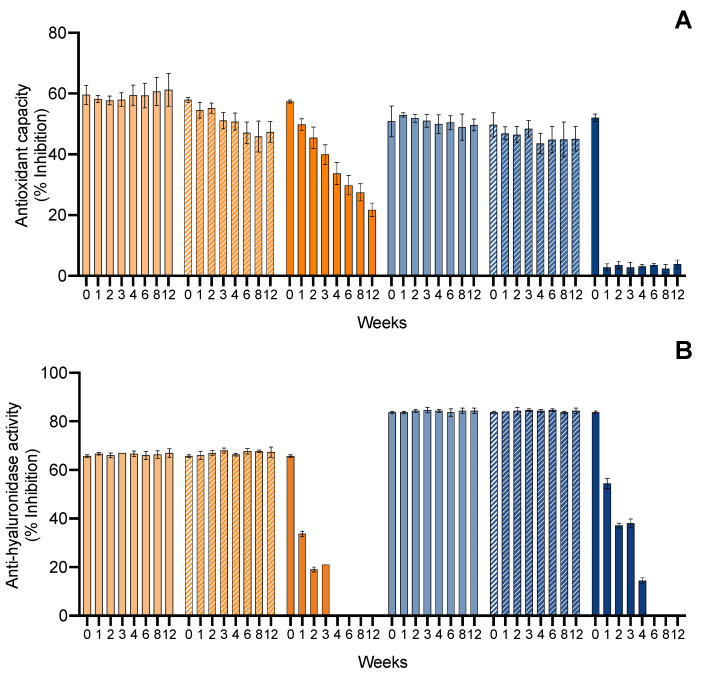
Effect of accelerated conditions (4 °C, 20 °C, and 40 °C) on the (**A**) ABTS^•+^ scavenging ability and (**B**) hyaluronidase inhibition activity (% inhibition; average ± standard deviation, *n* = 3) of ethanolic serum (E) and water serum (W). Colors of lines indicates different conditions: E 4 °C (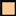); E 20 °C (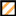); E 40 °C (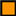); 4 °C (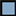); W 20 °C (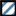); W 40 °C (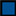).

Regarding the accelerated stability of serum pigments, the formulated serums were also subjected to different temperatures for 12 weeks and the pigment content, color change, and bioactive capacity were monitored. Results are shown in Figure 12 for ethanolic serum and Figure 13 for water serum. Due to the influence of antioxidant supplements in the bioactive capacity assays, only the control condition was evaluated for both serums (Figure 14).

The ethanolic serums subjected to 4 °C kept the carotenoid content with no statistical differences regardless of time and antioxidant supplementation. The ones subjected to 20 °C had a reduction in carotenoid content of about 30%, observed from weeks 2 to 12, regardless of the antioxidant supplementation; the supplementation of α-tocopherol and the mixture of antioxidants had a slower degradation. Finally, the serums subjected to 40 °C had a big reduction in carotenoid content from week 1 of about 50% and week 2 of about 50 mg g^−1^, representing a reduction of 75%.

When it came to color, the ethanolic serums subjected to 4 °C had no perceptible differences within the 12 weeks. The ones subjected to 20 °C had a big change in color from week 1 (ΔE ≈ 27.0), and even bigger from week 2 to 12 (ΔE ≈ 40.0). The optical correspondence goes from green to dark yellow, again due to the bleaching of chlorophyll. Finally, the serums subjected to 40 °C suffered a change in the color from week 1, with an ΔE > 40.0, corresponding to a change from bright green to brown and then to a muddy color.

Regarding the water serum, a similar pattern was found. The content was kept the same during the 12 weeks at 4 °C regardless of the antioxidant supplementation. At 20 °C all the serums had a reduction of 20% from week 4, with no further reduction. Thus, at 40 °C a linear decrease was observed in all serums from week 1 to week 4, with a reduction of 90% of total phycobiliprotein content. The changes directly affected the color: At 4 °C, no perceptible changes were found until week 4, when the ones supplemented with ascorbic acid had an ΔE = 10.0. The serums subjected to 20 °C had a linear color change with an increase in ΔE from week 1 (ΔE < 10.0), being more accentuated in week 4 (ΔE ≈ 30.0). The optical color equivalence changed from blue to greenish. Finally, the ones subjected to 40 °C had a big color change (blue to yellow/brown) from week 1 (ΔE ≈ 30.0), reaching ΔE > 50.0 by week 4.

Last, the bioactive capacity of the serum pigments (Figure 14) followed the trend found in total pigments and color. For the carotenoid serum, the antioxidant capacity was stable at 4 °C, had a modest decrease at 20 °C (1.2-fold), and lost the antioxidant capacity continuously at 40 °C, reaching only 21.75% of inhibition of ABTS^•+^ by 12 weeks. For the phycobiliprotein serum, the antioxidant capacity was stable at 4 °C and 20 °C, whereas at 40 °C it lost the antioxidant capacity by week 1 (<5%). Regarding the anti-hyaluronidase activity, both serums had a stable capacity at 4 °C and 20 °C. Both serums had an abrupt loss of bioactive capacity in the first month at 40 °C, losing their anti-hyaluronidase activity.

## 3. Discussion

As a vastly known producer of secondary metabolites such as mycosporine-like amino acids, alkaloids, amides, fatty acids, and peptides, cyanobacteria are an excellent source of natural products [17]. Moreover, pigments from cyanobacteria have been proposed as a highly bioactive group of compounds [9,10]. Because of their color and bioactive properties, these pigments are well known for their extremely appealing qualities for commercial application in food, feed, medicines, nutraceuticals, and cosmetics. Thus, when compared to carbohydrates, proteins, and lipids obtained from cyanobacteria, pigments emerged as the components with the highest market pricing, serving as the primary source of revenue for businesses [18]. Cyanobacterial pigments can be used either as pure compounds or as raw extracts. In the present study, the potential of *Cyanobium* sp. Extracts has reinforced that those extracts can be a functional source of bioactive compounds. Purification of compounds can account for up to 80% of the cost of production, and the use of extracts can be advantageous due to lower production costs and greater stability [19].

In this study, the cosmetic potential of *Cyanobium* sp. followed a continuous line of thought from the extract to a cosmetic ingredient and finally to an end product (skin serum). First, the two obtained pigment-target extracts were evaluated in terms of in vitro safety and cosmetic bioactive potential. As already mentioned, cyanobacteria are a unique and complex group of microorganisms that live in a wide range of environments. Their adaptation to these environments is linked to their ability to change their metabolisms and frequently produce secondary metabolites, which can be either an advantage as a bioactive ingredient or a disadvantage with toxic compounds. Cyanotoxins are thus a common group of compounds found in cyanobacteria [20]. These toxic compounds require careful consideration for both environmental impacts (blooms) and human health. Any food or ingredient derived from cyanobacterial biomass that is intended for human consumption must be thoroughly tested for the presence of these toxins. The results presented here suggest that *Cyanobium* sp. pigment-targeted extracts can be safe for cosmetic application. The strain has been also evaluated by Morone et al. [21], who found no cytotoxicity of ethanol (70%) in skin-related cell lines, although the authors tested relatively low concentrations (up to 100 μg mL^−1^). Moreover, Pagels et al. [13] also showed that ethanolic and successive water extracts had no cytotoxicity in HepG2 cells (liver) in concentrations up to 750 μg mL^−1^, although acetonic extracts showed cytotoxic effects in this cell line.

Regarding the bioactive potential, *Cyanobium* sp. extracts have been proposed as antioxidant and anti-inflammatory [13], and here, results showed the extracts were able to inhibit cosmetic-related enzymes: The phycobiliprotein-targeted extract was able to inhibit hyaluronidase and collagenase and the carotenoid-targeted extract was able to inhibit hyaluronidase. The anti-hyaluronidase capacity has been reported before in cyanobacteria extracts and purified compounds [22]. Yamaguchi et al. [23] showed that polysaccharide-targeted extracts from *Nostoc* spp. have high inhibitory potential with IC_50_ for hyaluronidase from 14.4 to 56.2 μg mL^−1^, depending on the species, whereas Yamaguchi and Koketsu [24] showed that a purified polysaccharide from *Nostochopsis lobatus* had an IC_50_ of 7.2 μg mL^−1^. Fujitani et al. [25] showed an ethanol-insoluble fraction from a water extract of *Arthrospira platensis* showed an IC_50_ of 150 μg mL^−1^. Furthermore, Montalvo et al. [26] showed that isolated peptides from *Arthrospira platensis* showed an IC_50_ from 920 to 1660 μg mL^−1^. Morone et al. [21] showed that ethanolic (70%) extract from *Tychonema* sp. and from another strain of *Cyanobium* sp. (LEGE 07175) had an IC_50_ for hyaluronidase of 182.7 and 208.4 μg mL^−1^, respectively. Moreover, a common ingredient in anti-aging products is green tea [15]. Here, as proof of concept, a water extract was prepared from *Camellia sinensis*, with an anti-hyaluronidase IC_50_ of 122.19 μg mL^−1^. The *Cyanobium* sp. extracts evaluated in this study showed an IC_50_ of 108.7 and 67.2 μg mL^−1^ for carotenoid-targeted and phycobiliprotein-targeted extracts, respectively, representing a powerful ingredient for anti-aging products.

When it came to collagenase inhibition, only *Cyanobium* sp. phycobiliprotein-targeted extract showed an IC_50_ of 582.8 μg mL^−1^. Collagenase inhibition has been studied in cyanobacteria to a smaller extent and focused on isolated compounds, with the examples of Montalvo et al. [26], who studied isolated peptides from *Arthrospira platensis* that showed an IC_50_ of 32.5 to 96.7 μg mL^−1^, and Tarasuntisuk et al. [27], who studied isolated mycosporine-2-glycine from *Aphanothece halophytica* that showed an IC_50_ for collagenase of ca. 115 μg mL^−1^.

The evaluation of *Cyanobium* sp. pigment-targeted extracts indicated that these extracts could be used as cosmetic ingredients; therefore, introducing these extracts into a vehicle would facilitate further product formulation. Linseed oil and glycerol were chosen as vehicles due to the current and approved used cosmetic ingredients, the compatibility with the extracts, and their green solvent label. The ingredients were subjected to hot–cold cycles and accelerated thermal stability. Overall, temperature similarly affected both ingredients. Lower temperatures (−20 °C ↔ 20 °C cycles and 4 °C and 20 °C treatments) preserved the pigment content, color, and antioxidant capacity during the study, except for the color change (from green to yellow) in the ethanolic ingredient (carotenoid-targeted) subjected to 20 °C for 12 weeks due to chlorophyll bleaching. However, in warmer temperatures (4 °C ↔ 40 °C cycles and 40 °C treatment), both ingredients were degraded, although it is notable that antioxidant supplements delayed that degradation—BHT and the antioxidant mixture for the ethanolic ingredient (carotenoid-targeted), and gallic acid and the antioxidant mixture for the water ingredient (phycobiliprotein-targeted). Pigments are thermo-sensitive compounds, and the stability can be reduced in higher temperatures; in addition, the vehicle itself can be less stable in such conditions. The stability of cyanobacterial pigments in cosmetic products has not been described, although a few studies on pigments have been found for food processing. Szterk et al. [28] showed a reduction in carotenoid content of 26.8% using linseed oil for a β-carotene beverage storage at 2 °C for 12 weeks, and the authors linked the loss of carotenoid content to the oxidation of the pigment and the oil. Noteworthy, the auto-oxidation of β-carotene at 30 °C can occur in about 30 h, and the supplementation of antioxidants such as BHT and α-tocopherol is required [29]. In this study, although BHT and the mixture of BHT and α-tocopherol delayed the effect of degradation, the degradation at higher temperatures was not avoided. Regarding phycobiliproteins, these natural pigments are sensitive to temperature, pH, humidity, and light. Galetović and Dufossé [30] evaluated the use of phycobiliproteins from *Nostoc* sp. as a colorant for dairy beverages and found that the isolated pigment was only stable in temperatures up to 21 °C (evaluated from 0 to 83 °C for 3 days), although it was stable during skim milk processing (138 °C for 4 s). In the food industry, preservatives such as citric acid, sodium chloride, calcium chloride, ascorbic acid, and benzoic acid are used to avoid the degradation of the products [30]. In this study, the addition of gallic acid was more advantageous than the addition of ascorbic acid, although no antioxidant was able to avoid degradation after one month at 40 °C. Mishra et al. [31] evaluated phycoerythrin stability at 0 and 35 °C for 45 days with the addition of commercial preservatives; at 0 °C the control treatment had a loss of 70% of phycoerythrin content, and at 35 °C a loss of 90%. The best preservative was citric acid, which led to a reduction of 50% at both temperatures.

Overall, the stability of *Cyanobium* sp. ingredients was satisfactory, and these ingredients were then included in a serum formulation. The chosen formulation was based on Chowjarean et al. [16], who had promising results in clinical trials of serum containing an extract of *Grammatophyllum speciosum* (vascular plant; orchid). Chowjarean et al. [16] evaluated the serum stability in 4 °C ↔ 40 °C cycles and 40 °C treatment, with positive results in terms of the bioactive compound stability (gastrodin; phenolic glycoside). Here, the results for *Cyanobium* sp. pigment serums were not as positive, but the stability in lower temperatures was indeed satisfactory. Similar to the equivalent ingredient, both serums were stable under lower temperatures (−20 °C ↔ 20 °C cycles and 4 °C treatment); however, at 20 °C, a small reduction in pigment content was observed (30% in carotenoids and 20% in phycobiliproteins), with a color change only in the ethanolic serum due to chlorophyll bleaching. Moreover, the abrupt degradation at 40 °C, which reduced the carotenoid content by 75% in two weeks and the phycobiliprotein content by 90% in one week, may require a lower shelf life or improvements in the formulation before commercialization. For example, the use of nanoparticles, as reviewed by Souto et al. [ref], who observed evidence of efficient delivery of natural extracts to cosmetic products when applied together with liposomes, chitosan/tripolyphosphate nanoparticles, and gold nanoparticles, among others, has already been applied in some brands/products (e.g., Chantecaille-Nano Gold Energizing Cream, Nanosomes^TM^).

Therefore, the requirement of low-temperature storage in skin serum is frequent in cosmetics, as it is common to find cosmetic fridges on sale (e.g., Skincare Mini Fridge, Cooluli, Brooklyn, NY, USA).

## 4. Materials and Methods

### 4.1. Cyanobacterial Biomass Source

*Cyanobium* sp. LEGE 06113 was obtained from Blue Biotechnology and Ecotoxicology Culture Collection (LEGE-CC). The cyanobacterium was grown in previously optimized conditions [12] for 14 days (10 days in white LED plus 4 days in red LED, aiming for maximum pigment content) with a light intensity of 200 μmol_photons_ m^−2^ s^−1^ and a light:dark cycle of 16:8 h. Blue Green medium (BG11) (Allen, 1968) was used as culture medium, with the addition of NaCl (10 g L^−1^), NaNO_3_ (3 g L^−1^), NaHCO_3_ (0.1 g L^−1^), and K_2_HPO_4_ (0.1 g L^−1^), and with pH set at 9.0 and kept constant with CHES-buffer (2 g L^−1^). Constant airflow was also assured at 0.75 L_air_ L^−1^ min^−1^. Biomass was harvested through centrifugation (10 min, 4000× *g*) and freeze-dried.

### 4.2. Pigment-Targeted Extracts

As previously optimized for *Cyanobium* sp. [13], the successive extraction using ethanol and then water led to two promising extracts rich in carotenoids and phycobiliproteins, being the selected extraction methodology for the present study. Two extracts were obtained from the freeze-dried biomass, an ethanolic one (carotenoid-targeted) and a water one (phycobiliprotein-targeted) [13]. For the ethanolic extract, cells were crushed using a Precellys Homogenizer bead beater (Bertin, France), using 250 mg of biomass in 3 cycles with 5 mL of ethanol (≥99.8%; 6 series of 8000 rpm for 30 s with 45 s of pause) and 670 mg of 0.1 mm beads to maximize cell disruption. Extracts were centrifuged (10 min 2000× *g*) and the supernatant was dried in a rotavapor. The remaining biomass was resuspended in 15 mL of water (phycobiliprotein-targeted extract), homogenized using a vortex, and centrifuged (10 min 2000× *g*). The supernatant was then freeze-dried. The quality of each extract was confirmed following the pigment content (30.7 ± 1.9 mg g^−1^ of carotenoids and 108.1 ± 7.9 mg g^−1^ of phycobiliproteins) as previously reported [13]. Both extracts were stored in low humidity (desiccator) in the dark until further analyses.

### 4.3. Extract Cytotoxicity

The cytotoxicity of both extracts was evaluated using 3-(4,5-dimethylthiazole-2-yl)-2,5-diphenyltetrazolium bromide (MTT) cytotoxicity assay in skin-related cell lines: a keratinocyte cell line (HaCat, ATCC), fibroblast cell line (3T3L1, ATCC), and endothelial cell line (hCMEC/D3) [21]. Cells were grown in DMEM Glutamax medium (Gibco, Waltham, MA, USA), supplemented with 10% (*v*/*v*) fetal bovine serum (Biochrom, Berlin, Germany), 1% Pen–Strep (Biochrom, Berlin, Germany) and 0.1% Amphotericin B (GE Healthcare, Chalfont St Giles, UK). Before extract exposure, cells were seeded in 96-well plates at densities of 2.5 × 10^4^ cells mL^−1^ (HaCat), 3.3 × 10^4^ cells mL^−1^ (3T3L1), and 1.0 × 10^5^ cells mL^−1^ (hCMEC/D3) and incubated for 24 h. Serial dilutions of the extracts (1.9 to 1000.0 μg mL^−1^) were prepared in DMEM with 1% DMSO, established as the maximum DMSO concentration not interfering with the assay. DMSO at 1% and 20% were used as the negative and positive control, respectively. Cells were exposed for 24 and 48 h. After each incubation time, 20 μL of 1 mg mL^−1^ MTT (Sigma-Aldrich) was added to each well and incubated for 3 h. Following incubation, the purple-colored formazan salts were dissolved in DMSO and the absorbance was read at 550 nm. The assay was performed in quadruplicate and independently repeated three times.

### 4.4. Enzymatic Activities

The cosmetic potential of extracts was evaluated by enzymatic assays: hyaluronidase, tyrosinase, collagenase, and elastase.

#### 4.4.1. Hyaluronidase

Hyaluronidase inhibition assay was determined as reported by Ferreres et al. [32]. First, 25 μL of extract dilutions (31.2 to 1000.0 μg mL^−1^) plus 175 μL of hyaluronic acid solution (0.7 mg mL^−1^ in water:buffer, 5:2 *v*/*v*, kept at 37 °C) were added to the reaction tube. The reaction was started by adding 25 μL of hyaluronidase (900 U/mL in NaCl 0.9%) and kept at 37 °C for 30 min. The reaction was stopped with 25 μL of disodium tetraborate (0.8 M), followed by subsequent heating for 3 min at 100 °C. After cooling to room temperature, 375 μL of DMBA solution (0.67 M) was added. The tubes were then incubated at 37 °C for 20 min and the absorbance of the colored product was measured at 560 nm. The enzymatic inhibition was calculated based on the values of 100% activity (using DMSO 10% instead of the extract) and 0% activity (using NaCl 0.9% instead of the enzyme). The assay was performed in triplicate.

#### 4.4.2. Tyrosinase

Tyrosinase inhibition assay was determined as reported by Adhikari et al. [33]. First, 10 μL of extract dilutions (31.2 to 1000.0 μg mL^−1^) plus 20 μL of tyrosinase (50 U mL^−1^) and 70 μL of phosphate buffer (50 mM, pH 6.5) were added to a 96-well plate and kept at 25 °C during 5 min. The reaction was started by adding 70 μL of the substrate (L-DOPA, 2.5 mM). Kojic acid was used as the positive control. The absorbance was measured at 0 and 15 min at 475 nm. The enzymatic inhibition was calculated based on the values of 100% activity (using DMSO 10% instead of the extract) and 0% activity (using buffer instead of the enzyme). The assay was performed in triplicate.

#### 4.4.3. Elastase

Elastase inhibition assay was determined as reported by Mota et al. [34]. First, 50 μL of extract dilutions (31.2 to 1000.0 μg mL^−1^) plus 87.5 μL of HEPES buffer (0.1 M with NaCl 0.5 M, pH 7.5), 10 μL of the substrate (N-succinyl-Ala-Ala-Ala p-nitroanilide, 1.12 mg mL^−1^), 70 μL of acetate buffer (200 mM, pH 5.5) and 2.5 μL of DMSO were added to a 96-well plate. The reaction was started by adding 30 μL of elastase (1 U mL^−1^) and kept at 37 °C for 10 min. The absorbance was then measured at 405 nm. The enzymatic inhibition was calculated based on the values of 100% activity (using DMSO 10% instead of the extract) and 0% activity (using buffer instead of the enzyme). The assay was performed in triplicate.

#### 4.4.4. Collagenase

Collagenase inhibition assay was determined as reported by Van Wart and Steinbrink [35] and modified by Andrade et al. [36]. First, 30 μL of extract dilutions (31.2 to 1000.0 μg mL^−1^) and 30 μL of collagenase (1 U mL^−1^) were added to a 96-well plate and kept at 37 °C for 15 min. The reaction was started by adding 120 μL of the substrate (FALGPA, 0.4 mM). The absorbance was read for 10 min at 345 nm. The enzymatic inhibition was calculated based on the values of 100% activity (using DMSO 10% instead of the extract) and 0% activity (using buffer instead of the enzyme). The assay was performed in triplicate.

### 4.5. Cyanobium sp. Cosmetic Ingredients

#### 4.5.1. Ingredient Vehicle

For better incorporation into cosmetic formulations, two ingredients (vehicle plus extract) were proposed; the ethanolic ingredient contained the ethanolic extract resuspended in linseed oil and the water ingredient contained the water extract resuspended in 80% glycerol. Both extracts were kept in a concentration of 5 mg g^−1^.

#### 4.5.2. Ingredient Characterization

The *Cyanobium* sp. ingredients were characterized following ISO/TR 18811/2018 and European Regulation EC 1223/2009 in terms of color, pH, phase separation, viscosity, density, conductivity, total pigments, and bioactive capacity (antioxidant and anti-hyaluronidase). The characterization assay was performed in triplicate batches of 50 mL of each ingredient (without antioxidant supplementation) and all parameters were analyzed in triplicate for each ingredient replicate.

#### 4.5.3. Color

Color measurements were performed using a CR-400 colorimeter (Konica Minolta, Japan) with an aperture of 8 mm at standard illuminate D65 using the CIE 1976 (L*, brightness; a*, redness; b*, yellowness). The CIE system uses a three-dimensional colorimetric measurement system: L* values represent the color’s brightness, a* values represent the red–green content, and b* values represent the yellow–blue content. Color changes (ΔE) determine the three-dimensional color space and are calculated as:

ΔE = [(ΔL*)^2^ + (Δa*)^2^ + (Δb*)^2^]^1/2^ (1)

The human perception chart was followed as not perceptible (ΔE ≤ 1.0), perceptible through close observation (1.0 < ΔE ≤ 2.0), perceptible at a glance (2.0 < ΔE ≤ 10.0), colors are more similar than different (10.0 < ΔE ≤ 50.0), and colors are completely different (ΔE > 50.0) [37].

#### 4.5.4. pH and Conductivity

pH and conductivity were measured using an HQ40D digital two-channel multimeter (Hach, Loveland, CO, USA), using an Intellical™ PHC101 pH probe (Hach) and an Intellical™ CDC401 conductivity probe. The noteworthy pH of linseed oil is relative, as the calibration is performed using aqueous buffers.

#### 4.5.5. Phase Separation

*Cyanobium* sp. ingredients were centrifuged (15 min 2000× *g*) to evaluate phase separation [38].

#### 4.5.6. Viscosity

Viscosity was measured using a Zahn cup viscosimeter with an aperture of 2.74 mm and a working volume of 44 mL (Baoshishan, China).

#### 4.5.7. Total Pigments

Pigments were quantified spectrophotometrically. For the ethanolic ingredient, total carotenoids were quantified following Zavřel et al. [39], diluting the ingredient in methanol in a final concentration of 0.5 mg mL^−1^. For the water ingredient, total phycobiliproteins were quantified following Bennett and Bogobad [40], diluting the ingredient in water in a final concentration of 0.5 mg mL^−1^. The results were expressed in milligrams per gram of dry extract (mg g^−1^).

#### 4.5.8. Bioactive Capacity

The bioactive capacity of the *Cyanobium* sp. ingredients was evaluated in terms of antioxidant capacity and anti-hyaluronidase activity. The antioxidant capacity was evaluated via the ABTS^•+^ assay [41] with some modifications—the assay was performed in triplicate in a 96-well plate. A total of 63 μL of the sample was added to 180 μL of ABTS reagent and gently shaken. The reaction occurred in the dark for 6 min and the plate was read at 734 nm; the final concentration of the ingredient was 250 μg mL^−1^. The anti-hyaluronidase activity was evaluated as described before, with a final concentration of the ingredient of 250 μg mL^−1^.

#### 4.5.9. Antioxidant Supplementation and Compatibility

The formulation containing antioxidants was evaluated to keep the color and pigment content in both extracts. The ethanolic ingredient was supplemented with 1 mg g^−1^ α-tocopherol, BHT (butylated hydroxytoluene), or a mixture of both (1:1, *w*/*w*), whereas the water ingredient was supplemented with 1 mg g^−1^ ascorbic acid, gallic acid, or a mixture of both (1:1, *w*/*w*). Both ingredients were also evaluated without antioxidant supplement (control) and the vehicle without extract (80% glycerol and linseed oil).

#### 4.5.10. Ingredient Hot–Cold Stability

To assess the physical stability of the *Cyanobium* sp. ingredients, a hot–cold stability study was carried out, following ISO/TR 18811/2018 and European Regulation EC 1223/2009. The extract ingredients with and without antioxidants were subjected to two heating–cooling cycle assays in 24-well plates with a working volume of 1.7 mL: (1) 7 cycles of 24 h at 4 ± 2 °C followed by 24 h at 40 ± 2 °C, and (2) 7 cycles of 24 h at −20 ± 2 °C followed by 24 h at 20 ± 2 °C. The color, total pigments, and bioactive capacity (antioxidant and anti-hyaluronidase) were evaluated at the beginning and after each cycle. The stability assay was performed in triplicate in all parameters.

#### 4.5.11. Accelerated Ingredient Stability

Long-term stability and determination of the period-after-opening (PAO) were assessed by an accelerated stability test following ISO/TR 18811/2018 and European Regulation EC 1223/2009. The *Cyanobium* sp. ingredients with and without antioxidants were subjected to three different temperatures (40 ± 2, 4 ± 2, 20 ± 2 °C) for 12 weeks in 24-well plates with a working volume of 1.7 mL. Color, total pigments, and bioactive capacity (antioxidant and anti-hyaluronidase) were evaluated at nine timepoints of the assay (W0, W1, W2, W3, W4, W6, W8, W10, W12). The stability assay was performed in triplicate and all parameters were analyzed in triplicate for each ingredient replicate.

### 4.6. Serum Formulation

#### 4.6.1. Formulation

To evaluate the possible application of *Cyanobium* sp. ingredients, a basal serum in water was formulated following Chowjarean et al. [16] composed of PEG 400 (12%), Aristoflex AVC (Clariant, Switzerland) (0.5%), Microcare PHC (1%), and extract vehicle (3%). The extracts were added to the vehicle for a final concentration in the serum of 5 mg g^−1^.

#### 4.6.2. Formulation Characterization

The *Cyanobium* sp. serum formulations were characterized following ISO/TR 18811/2018 and European Regulation EC 1223/2009 in terms of color, pH, phase separation, viscosity, density, conductivity, total pigments, and bioactive capacity (antioxidant and anti-hyaluronidase). The characterization assay was performed in triplicate batches of 50 mL of serum formulation (without antioxidant supplementation) and all parameters were analyzed in triplicate for each serum replicate.

#### 4.6.3. Serum Hot–Cold Stability

To assess the physical stability of the *Cyanobium* sp. serum formulations, a hot–cold stability study was carried out, following ISO/TR 18811/2018 and European Regulation EC 1223/2009. The formulations were subjected to two heating–cooling cycle assays in 24-well plates with a working volume of 1.7 mL: (1) 7 cycles of 24 h at 4 ± 2 °C followed by 24 h at 40 ± 2 °C, and (2) 7 cycles of 24 h at −20 ± 2 °C followed by 24 h at 20 ± 2 °C. Color, total pigments, and bioactive capacity (antioxidant and anti-hyaluronidase) were evaluated at the beginning and after each cycle. The stability assay was performed in triplicate in all parameters.

#### 4.6.4. Accelerated Serum Stability

Long-term stability and determination of the period-after-opening (PAO) were assessed by an accelerated stability test following ISO/TR 18811/2018 and European Regulation EC 1223/2009. The *Cyanobium* sp. serum formulations were subjected to three different temperatures (40 ± 2, 4 ± 2, 20 ± 2 °C) for 12 weeks in 24-well plates with a working volume of 1.7 mL. Color, total pigments, and bioactive capacity (antioxidant and anti-hyaluronidase) were evaluated at nine timepoints of the assay (W0, W1, W2, W3, W4, W6, W8, W10, W12). The stability assay was performed in triplicate and all parameters were analyzed in triplicate for each ingredient replicate.

### 4.7. Statistical Analysis

Statistical analyses were performed using GraphPad Prism v.8 software (GraphPad, San Diego, CA, USA). IC values for enzymatic activity were calculated through curve spline interpolation. Each data set’s homoscedasticity was verified by the Cochran test. One-way ANOVA was used for enzymatic inhibition, cytotoxicity, and physical and chemical characterization. Whenever significant differences were detected, post hoc multiple comparisons were made, for cytotoxicity using Dunnett’s test to identify differences between control and Tukey´s test for enzymatic inhibition and physical–chemical characterization. The significance level in all analyses was 95% (*p* < 0.05). For stability assays, a two-way ANOVA was performed, and whenever significant differences were detected, post hoc multiple comparisons were made using Tukey’s test to identify differences for the conditions and time.

## 5. Conclusions

The cosmetic potential of pigment-targeted extracts from *Cyanobium* sp. was proposed and evaluated in three steps: extract, ingredient, and product. The extract showed no cytotoxic effects in skin-related cell lines, with a high anti-hyaluronidase capacity in both extracts and an anti-collagenase effect in the water extract. Moreover, both extracts were stable as ingredients and products (skin serum) at low temperatures (−20 °C ↔ 20 °C cycles, and 4 °C and 20 °C treatments) and it was possible to keep the pigment content and antioxidant capacity stable during the testing period, whereas at higher temperatures (40 °C) the product degraded in a week. Furthermore, because of their in vitro bioactive capacity and stability, both extracts can be potential ingredients for cosmetic uses (anti-aging), with relatively simple formulation and storage. Finally, the approach used in this study, by evaluating extract, ingredient, and product, gives a much wider overview of the real applicability of the extracts within the cosmetic industry, highlighting not only the potential of *Cyanobium* sp. extracts as a cosmetic ingredient but also the use of other sources of cyanobacteria apart from the ones already used in the industry.

## 6. Patents

This work has formed the basis for a patent application—Portuguese Provisional Patent Application No. 117951—in which the authors are inventors.

## Figures and Tables

**Table 1:** Physical and chemical characterization of *Cyanobium* sp. ingredients.

Parameter	Ethanolic Ingredient	Water Ingredient
pH	4.71 ± 0.06	7.78 ± 0.05
Phases	1	1
Viscosity (cst)	32.85 ± 1.27	74.55 ± 4.88
Density (g cm^−3^)	0.81 ± 0.01	0.98 ± 0.02
Conductivity (μS cm^−1^)	0.41 ± 0.08	7.36 ± 0.06
Color ^+^		
L*	35.98 ± 2.12	36.93 ± 1.09
a*	−29.33 ± 1.18	−22.72 ± 1.32
b*	45.30 ± 3.16	−8.58 ± 0.85
Optical correspondence	Green	Blue
Antioxidant capacity (ABTS^•+^ IC_50_, mg mL^−1^)	140.69 ± 6.31	180.93 ± 7.91
Anti-hyaluronidase activity (IC_50_, mg mL^−1^)	115.37 ± 10.33	79.41 ± 6.43
Total pigments * (mg g^−1^)	181.83 ± 8.17	505.13 ± 8.18

* Carotenoids for the ethanolic ingredient and phycobiliproteins for the water ingredient. ^+^ L*: brightness; a*: redness; b*: yellowness.

**Table 2:** Physical and chemical characterization of *Cyanobium* sp. serum formulations.

Parameter	Ethanolic Serum	Water Serum
pH	7.42 ± 0.06	7.47 ± 0.05
Phases	2	1
Viscosity (cst)	28.46 ± 1.45	26.95 ± 1.21
Density (g cm^−3^)	1.09 ± 0.02	0.96 ± 0.01
Conductivity (μS cm^−1^)	1714 ± 41	1613 ± 55
Color ^+^		
L*	35.11 ± 2.02	39.93 ± 2.17
a*	−30.47 ± 2.94	−25.77 ± 2.10
b*	51.82 ± 4.39	−9.11 ± 0.54
Optical correspondence	Green	Blue
Antioxidant capacity (ABTS^•+^ IC_50_, mg mL^−1^)	186.12 ± 10.42	205.18 ± 13.16
Anti-hyaluronidase activity (IC_50_, mg mL^−1^)	135.53 ± 11.23	85.43 ± 3.40
Total pigments * (mg g^−1^)	175.34 ± 12.72	497.38 ± 10.82

* Carotenoids for the ethanolic serum and phycobiliproteins for the water serum. ^+^ L*: brightness; a*: redness; b*: yellowness.

## Data Availability

Not applicable.
